# A Mesh Space Mapping Modeling Method with Mesh Deformation for Microwave Components

**DOI:** 10.3390/mi14091783

**Published:** 2023-09-17

**Authors:** Shuxia Yan, Chenglin Li, Mutian Li, Zhimou Li, Xu Wang, Jian Wang, Yaocong Xie

**Affiliations:** 1 School of Electronics and Information Engineering, Tiangong University, Tianjin 300387, China; yanshuxia@tiangong.edu.cn (S.Y.); 2131070907@tiangong.edu.cn (C.L.); jiaxuwang@tju.edu.cn (X.W.); 2 School of Microelectronics, Tianjin University, Tianjin 300072, China; mutianli@tju.edu.cn; 3 Qingdao Institute for Ocean Technology, Tianjin University, Qingdao 266200, China; 4 School of Information and Control Engineering, Qingdao University of Technology, Qingdao 266520, China

**Keywords:** space mapping, modeling, mesh deformation, microwave components

## Abstract

In this study, a low-cost space mapping (SM) modeling method with mesh deformation is proposed for microwave components. In this approach, the coarse-mesh model with mesh deformation is developed as the coarse model, and the fine-mesh model is simulated as the fine model. The SM technique establishes the mapping relationship between the coarse-mesh model and the fine-mesh model. This approach enables us to combine the computational efficiency of the coarse model with the accuracy of the fine model. The automatic mesh deformation technology is embedded in the coarse model to avoid the discontinuous change in the electromagnetic response. The proposed model consisting of the coarse model and two mapping modules can represent the features of the fine model more accurately, and predict the electromagnetic response of microwave components quickly. The proposed mesh SM modeling technique is applied to the four-pole waveguide filter. The value for the training and test errors in the proposed model is less than 1%, which is lower than that for the ANN models and the existing SM models trained with the same data. Compared with HFSS software, the proposed model can save about 70% CPU time in predicting a set of 100 data. The results show that the proposed method achieves a good modeling accuracy and efficiency with few training data and a low computational cost.

## 1. Introduction

In the field of semiconductor design, the design of passive high-frequency devices mainly relies on electromagnetic (EM) physical models. Physical EM models in simulation software describe the behavior of microwave components by analyzing the transmission characteristics of the EM wave. Mathematical equations, such as Maxwell’s system of equations, are established to represent the relationship between the structure parameters and the responses of the device. The computational process requires extensive numerical calculations to provide high-fidelity results, reducing the efficiency of device design [1]. In particular, when the geometric parameters need to be adjusted repeatedly, the device design cycle is greatly extended. Efficient surrogate models are used in device design to address time consumption [2,3]. The traditional modeling method usually develops and improves the model through continuous testing and repeated correction, which is a slow trial-and-error process. As the structural complexity of microwave devices increases, and product performance requirements improve, the traditional modeling method cannot meet the modeling requirements. To reduce the computational cost, and adapt to the new modeling requirements, modeling methods with a high efficiency and high fidelity are being studied [4].

Space mapping (SM) [5,6,7,8,9,10,11,12,13,14] technology has been widely used to solve the growing computational challenges in the field of microwave modeling. The SM algorithm assumes the existence of coarse models [5], and uses empirical functions [15] or equivalent circuits [16,17,18] as prior knowledge to assist in the modeling process of microwave devices. The SM combines the high computational efficiency of the coarse model and the high fidelity of the EM fine model [19]. A neural SM approach for nonlinear device modeling was proposed in [20], which adds a dynamic neural network to an existing equivalent circuit model to compensate for capacitance effects that are missed in the given model. The SM technique is used to map the computational efficiency of the EM single-physics model to the accuracy of the multi-physics model, which achieves a good multi-physics parametric modeling accuracy with less multi-physics training data and a lower computational cost [21]. The SM method in [22] speeds up the model development process by developing separate mappings for different expenditures within an existing device model, allowing different EM behaviors of the device to be mapped individually.

However, the coarse models represented by empirical functions or equivalent circuits are not always accurate enough [23]. In most three-dimensional device structures (e.g., antennas, filters, etc.), some empirical formulas are too complex, or some equivalent circuits are not applicable, rendering the coarse model invalid. In the physical EM model, the continuous structure is transformed into discrete mesh cells via discretization. EM models with different numbers of mesh cells are used in the surrogate model. Geometric models with coarse mesh are chosen as the coarse model in the SM method [24,25]. In the process of building the coarse model with existing modeling software, the coarse mesh is usually generated using the mesh adaptive method. When the values of the geometric parameters change, the coarse meshes need to be re-generated [26]. As the coarse-mesh model is still essentially a three-dimensional structure, its simulation time is longer than the equivalent circuit model, which increases the SM model modeling time. In addition, the existing mesh methods lead to the discontinuity and non-convergence of the electromagnetic response. Therefore, the method of building the coarse model with coarse mesh still needs to be improved.

In this paper, we propose a modeling approach combining the coarse-mesh model and mapping modules. In this way, SM can be performed even if the equivalent circuit model is not available. We also incorporate the mesh deformation technique to accelerate the SM process, which improves the modeling efficiency by reducing repetitive EM simulation. In addition, an automatic training method is proposed to obtain a high-accuracy model with few EM data. The effectiveness of the coarse-fine mesh modeling technique is validated by examples of a four-pole waveguide filter with tuned elements.

## 2. Mesh SM Modeling Method with Mesh Deformation

In this paper, the mesh SM modeling technique for microwave components is proposed to accelerate modeling processes. A fine-mesh model with more meshes has a more accurate response and high computational costs. A coarse-mesh model with fewer meshes has faster computation speeds and a low accuracy. We define the fine-mesh model as the fine model, while choosing the coarse-mesh model with mesh deformation as the coarse model. Fine models provide more accurate results with high computational costs, while coarse models have faster computation speeds with a low accuracy. Mapping modules are introduced to represent the relationship between the fine and coarse model, then to form the new surrogate model. The proposed surrogate model with a high accuracy and fast running speed is established to replace the time-consuming fine model in circuit design. The modeling process is presented in Section 2.1, the mesh deformation in Section 2.2, the SM structure in Section 2.3, and the more detailed model training process in Section 2.4.

### 2.1. Mesh SM Modeling Process

**Figure 1:**
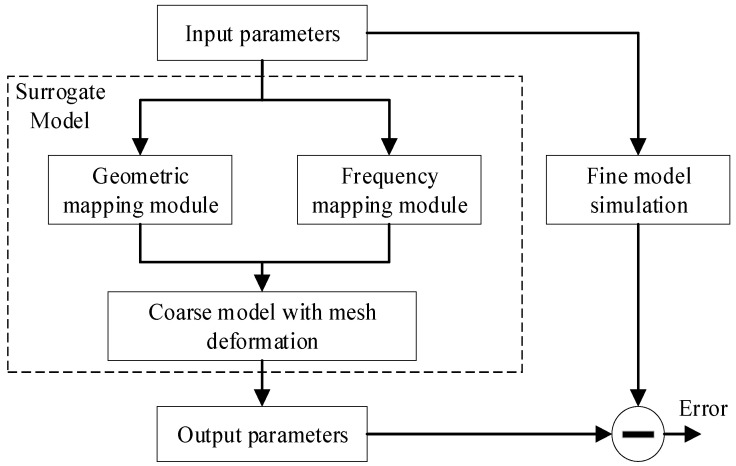
Schematic of the proposed modeling method.

We proposed a surrogate model using the mesh SM technique. Compared with the fine model, which is accurate but time-consuming, the coarse model, which represents fine characteristics approximately, is simpler and faster. The proposed surrogate model in this paper utilizes the coarse model, with mesh deformation as knowledge. Mapping modules are incorporated into the coarse model to reduce discrepancies between the fine model and the coarse model. The overall structure of the SM model is illustrated in Figure 1.

The proposed surrogate model contains the coarse model and two mapping modules. The geometric and frequency parameters are the inputs of the model. If the same input values act on both the coarse model and the fine model, then the output responses of the coarse model are inconsistent with those of the fine model. The input parameters are adjusted via the mapping modules before loading into the coarse model. The geometric mapping module develops the relationship of geometric parameters between the coarse model and the fine model, and the frequency mapping module represents the relationship of frequency parameters between the two models. The coarse model is an EM model with coarse meshes, which incorporate mesh deformation to maintain the continuity of the model response. The adjusted geometric and frequency parameters act on the coarse model. For the initial mapping modules, the error, which represents the difference in output parameters between the coarse model and the fine model, is larger. The training process is performed to modify the weights of the two mapping modules, meaning that the response of the coarse model matches that of the fine model. During the training process, the response of the surrogate model changes with the weight vectors, which makes the error gradually smaller. When the error is lower than the threshold value, the surrogate model is consistent with the EM response of the fine model for different geometric and frequency parameters.

### 2.2. Mesh Deformation Technique of the Coarse Model

Meshing is an important part of the finite element method (FEM) [27], which divides the continuous structure into discrete finite element cells. Meshes need to be regenerated when the design parameters change. New meshes result in discontinuous responses to the model. In this paper, the coarse model is constructed via incorporating mesh deformation into the coarse mesh. Mesh deformation technique based on the radial basis function (RBF) [28] can generate high-quality finite element meshes quickly and automatically. In mesh deformation, the RBF can be used to interpolate the control points of the original mesh to produce new meshes with deformation effects. Vector $g_{c}$ is employed to represent the geometrical parameters of the coarse model. When $g_{c}$ changes slightly, the corresponding mesh nodes shift. After the incorporation of the mesh deformation technique, the number of mesh nodes and the connectivity information of the overall model are unchanged. Therefore, continuous variation in the EM responses can be guaranteed.

To smoothly calculate the displacement of each node inside the mesh, we can abstract the mesh deformation problem as an interpolation problem. In mesh deformation, the displacement values of the interior nodes can be calculated via the interpolation principle. The input variable m is predicted or calculated. The displacement values $m_{i}$ on the boundary are known, and the index of the boundary nodes is denoted by the variable i. The RBF interpolation method defines the deformation function as the linear combination of basis functions φ. In the proposed method, we utilized the thin plate spline [29] as the basis function. $\varphi_{i}$ is an RBF depending on the Euclidean distance between m and $m_{i}$, which is expressed as

(1) $$\varphi_{i}(m)=\varphi (||m-m_{i}||)$$

For three-dimensional mesh deformation, the mesh nodes are divided into two parts. We define the number of the boundary nodes as $N_{b}$, and the number of the interior nodes as $N_{v}$. We denote the three-dimensional coordinates of the *i*-th mesh node as $d_{i}$. When the geometrical dimensions change, mesh nodes are displaced due to the mesh deformation function. Therefore, the new location $d_{i}$ of the *i*-th mesh node after the mesh deformation can be expressed as

(2) $$d_{i}(g_{c})=\sum_{i=1}^{N_{b}}W_{i}(g_{c})\varphi_{i}(m)$$

where $W_{i}$ denotes the weight of the RBF. The affine motion is the transformation of a geometric object to another location via linear transformation. We characterize the mesh deformation using linear trigonometric polynomial space. By adjusting the coefficients $q_{j}$, we can control each node of the mesh to find the optimal affine motion, including translation, rotation, and scaling. Thus, an additional term is added to (1), and the expression of $d_{i}$ can be formulated as

(3) $$d_{i}(g_{c})=\sum_{i=1}^{N_{b}}W_{i}(g_{c})\varphi (||m-m_{i}||)+\sum_{j=1}^{4}q_{j}(x+y+z+1)$$

where $q_{j}$ is the linear trigonometric polynomial weighting coefficient representing the coupling between coordinates of different latitudes. x, y, z, and 1 are the basis for the space of linear ternary polynomials. For any mesh node, we utilize (3) to calculate the new location by taking the Euclidean distance between m and $m_{i}$ as input. $W_{i}$ and $q_{j}$ need to be reconstructed during each SM iteration when geometrical parameters change. The total number of mesh nodes is defined as $N_{t}$, where $N_{t}=N_{b}+N_{v}$. Assuming that $D_{k}$ denotes the three-dimensional coordinates of all mesh nodes, which is $D_{k}=[d_{1},d_{2},...,d_{N_{t}}]$, as $D_{k}$ changes continuously with $g_{c}$, the EM response of the coarse mesh model can continuously vary according to the FEM.

This technique achieves the flexible deformation of the mesh domain by applying the RBF interpolation method to the relative mesh nodes. In the process of RBF mesh deformation, the initial stage involves the selection of suitable RBF types and parameters for application to the mesh nodes. Subsequently, the entire mesh undergoes deformation through the adjustment of the RBF weight coefficients. The deformed nodes are then reconnected to yield the deformed mesh.

The mesh deformation proposed in this paper is the simple deformation accomplished on the original meshing. After the embedding of the mesh deformation technique, the EM response of the coarse model changes continuously with the geometric dimensions. The proposed method speeds up the convergence of the algorithm, reduces the number of redundant iterations, and improves the accuracy of the SM algorithm.

### 2.3. Mesh SM Structure

**Figure 2:**
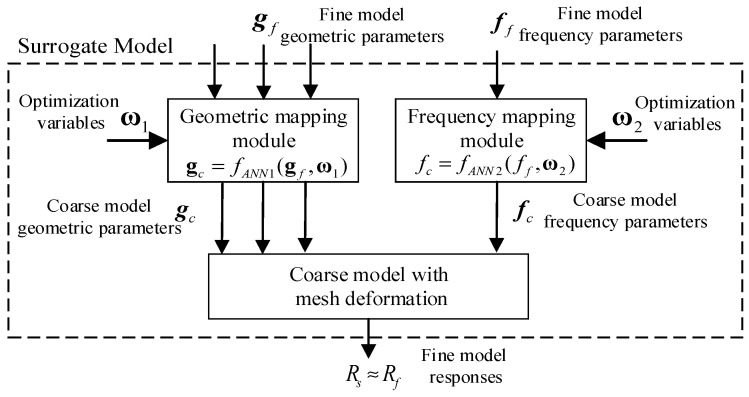
The structure of the proposed model.

Reducing the response gap between the coarse and fine models, two mapping modules are introduced into the surrogate model. Two mapping modules establish the relationship between the coarse and fine models, meaning that the complex fine model is replaced with the simplified coarse model to speed up computation. The surrogate model, as accurate as the fine model, is computationally efficient. Figure 2 illustrates the structure of the proposed surrogate model.

The inputs of both the fine model and the surrogate model have two parts: the geometric parameters represented by $g_{f}$ and the frequency parameters represented by $f_{f}$. Assuming that the vector of the fine model response is $R_{f}$, the response vector of the surrogate model is denoted as $R_{s}$. To ensure that the coarse model outputs are consistent with those of the fine model, the geometric and frequency parameters are adjusted via two mapping modules. The parameters $g_{c}$ and $f_{c}$ are vectors of the geometric and frequency parameters of the coarse model, respectively. The mapping relationship of input parameters between the coarse model and the fine model is denoted, respectively, as:

(4) $$g_{c}=f_{ANN1}(g_{f},\omega_{1})$$

and

(5) $$f_{c}=f_{ANN2}(f_{f},\omega_{2})$$

where $f_{ANN1}$ and $f_{ANN2}$ are mapping functions that can be implemented in a variety of ways. Simple linear functions, such as the linear polynomial, or complex nonlinear functions, such as the multilayer perceptron (MLP), can represent the mapping relationship. The vectors $\omega_{1}$ and $\omega_{2}$ are weight parameters in $f_{ANN1}$ and $f_{ANN2}$, adjusted during the training process. After performing the input SM modeling from $g_{f}$ to $g_{c}$, the formulation of mesh deformation for the proposed SM model is derived as

(6) $$d_{i}(g_{f},\omega_{1})=\sum_{i=1}^{N_{b}}W_{i}(f_{ANN1}(g_{f},\omega_{1}))\varphi (||m-m_{i}||)+\sum_{j=1}^{4}q_{j}(x+y+z+1)$$

To develop the proposed overall SM model, the response vector of the coarse model can be denoted as $R_{c}$, which depends on the input vectors $g_{c}$ and $f_{c}$, i.e., $R_{c}(g_{c},f_{c})$. The response of the proposed model with geometric and frequency parameters is defined as:

(7) $$R_{s}(g_{f},f_{f},\omega_{1},\omega_{2})=R_{c}(f_{ANN1}(g_{f},\omega_{1}),f_{ANN2}(f_{f},\omega_{2}))$$

The weight parameters $\omega_{1}$ and $\omega_{2}$ are adjusted in the training process, meaning that the outputs of the surrogate model match those of the fine model when using the same geometric and frequency variables.

### 2.4. Mesh SM Training Process

The coarse model is constructed through meshing the geometric structure of the coarse-mesh model with mesh deformation. A surrogate model is then established via combining the coarse model and the mapping modules. The mapping modules connect the coarse- and fine-mesh models, and the mapping functions are expressed in Section 2.3. The proposed mesh SM training process has two stages.

In the first stage, we build the coarse model, which reduces complexity and computation, using meshing and mesh deformation techniques. To allow the whole training process to be implemented in Neuromodelerplus [30] software, and speed up the training process, an MLP is employed to represent the characteristics of the coarse model. The MLP is trained to minimize the training error as much as possible. The development process of the coarse model stops when the test error falls below a given threshold θ. Otherwise, the MLP structure is rebuilt.

In the second stage, two mapping modules developing the relationship between the coarse and fine models are added to the coarse model. To ensure that the error does not increase after the joining of the mapping modules, firstly, two unit mapping functions are established with the unit data, meaning that the outputs are equal to the inputs. The characteristics of the surrogate model with unit mapping networks are as same as those of the coarse model. Then, the design of experiments (DOE) sampling method generates data for the surrogate model training [31]. The weights of the two mapping modules are adjusted to represent the relationship between the coarse and fine models accurately. After training, the surrogate model with accurate responses can replace the fine model.

**Figure 3:**
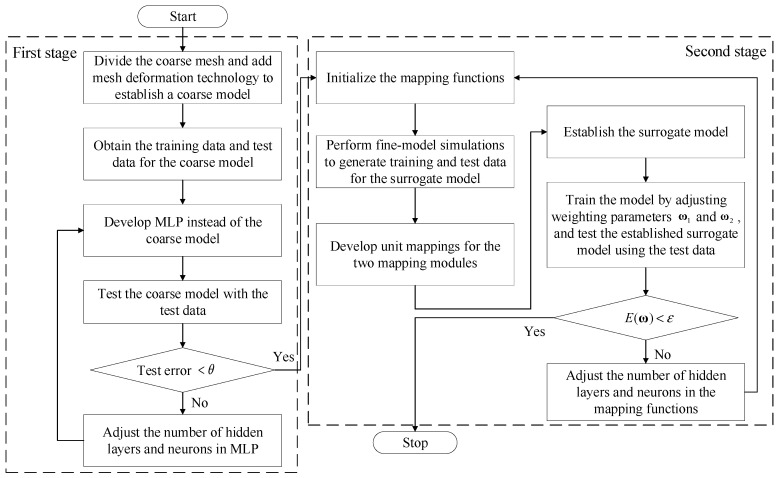
Flowchart of the proposed surrogate modeling process.

Data for training and testing the surrogate model are generated via simulating the fine model. The weight vectors $\omega_{1}$ and $\omega_{2}$ in the mapping functions $f_{ANN1}$ and $f_{ANN2}$ are adjusted to minimize the error of the responses between the surrogate model and the fine model. The error E(ω) is expressed as

(8) $$E(\omega )=\frac{1}{2}{\sum_{k=1}^{p}\sum_{l=1}^{q}\left\|R_{s}(g_{f}^{k},f_{f}^{l},\omega_{1},\omega_{2})-d_{k,l}\right\|}^{2}$$

where $R_{s}$ and d represent the EM response vector of the surrogate model and the fine model. k and l are the number of training sample data and the selected frequency points, respectively. That is, $k={\left[1,2,\ldots ,p\right]}^{T}$ and $l={\left[1,2,\ldots ,q\right]}^{T}$, the maximum number of training sample data and selected frequency points, are denoted by p and q. The established surrogate model is tested with test data. The modeling process terminates until the test error E(ω) satisfies E(ω)<ε, where ε is the defined threshold. The appropriate weight vectors can be obtained through this process, and the characteristics of the surrogate model are the same as those of the fine model. The flowchart of the proposed surrogate modeling process is shown in Figure 3.

## 3. Example Verification

**Figure 4:**
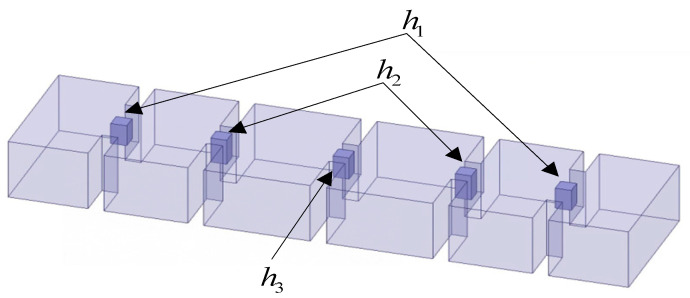
The structure of a four-pole waveguide filter.

A four-pole waveguide filter, with tuning elements acting as pillars for square sections placed in the center of each cavity and each coupling window, is used in this section. The structure can be seen in Figure 4. The fine and coarse models use different numbers of meshes for EM simulations. The fine model uses more mesh cells (about 330,000 cells), while the coarse model uses fewer mesh cells (about 4400 cells). To acquire the response of the fine model, the mesh adaptive process generates the fine mesh by refining the mesh in the HFSS software. The design parameters mentioned here are $g_{f}={\left[h_{1},h_{2},h_{3}\right]}^{T}$, which represent the parameters of the shape and size of the structure. The frequency $f_{f}$ is an additional input for the fine model. As illustrated in Figure 4, the heights of the tuning columns in the coupling window are represented by $h_{1}$, $h_{2}$, and $h_{3}$, which are chosen as geometric variables. Through the adjustment of these design variables, the structural parameters are changed in the EM simulation, affecting the model response. For surrogate model modeling, the fine-mesh model with a mesh adaptive process is operated to obtain accurate model data.

**Figure 5:**
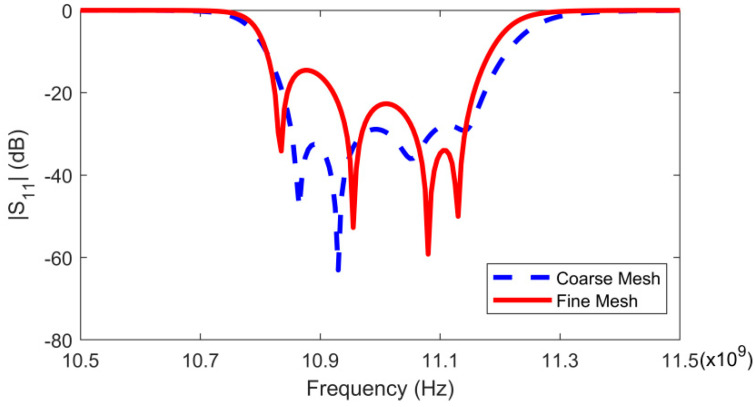
Response comparison between the coarse and fine models with the same geometric parameters.

The coarse model is constructed with the same three geometric variables $g_{c}={\left[h_{1c},h_{2c},h_{3c}\right]}^{T}$. For the coarse model, the frequency $f_{c}$ serves as an additional input. Fine model simulations with the entire mesh information are performed in HFSS to generate the training data and test data for different geometric and frequency parameters. In this example, the coarse mesh with mesh deformation is employed to construct the coarse model with geometric parameters used as variables. The geometry of the coarse model is meshed using Gmsh software. To avoid discontinuous change in the EM response, mesh deformation was added in the construction of the coarse model using MATLAB software. To allow the whole training process to be implemented in Neuromodelerplus software, and to speed up the training process, MLPs were employed to represent the characteristics of the coarse model. The accuracy of the coarse model is lower than that of the fine model, and the responses between the coarse and fine models are not completely consistent. Figure 5 shows the comparison between the coarse model response and the fine model response with the same geometric parameters. To match the output of the coarse model with that of the fine model, mapping networks are employed in this paper to adjust the input variables of the coarse model.

The mesh deformation incorporated in the coarse model constantly calls MATLAB software during the training process, which increases the training time greatly. To reduce repeated calls to MATLAB, we chose to use MLP with 50 hidden neurons instead of coarse models. In the EM domain, the DOE method is applied as a sampling technique to generate data for the construction of the coarse model. The simulation data for the coarse model were constructed with the geometric parameters as variables. Table 1 shows the specific values of the training and test data for the coarse model modeling. We used nine levels (81 sets of data) of DOE to define the training data samples, and eight levels (64 sets of data) of DOE for the test data. Neuromodelerplus software was utilized to train the MLP instead of the coarse model. The MLP’s training error is 0.159% with the 81 sets of training data. The MLP’s test error is 0.134% with the 64 sets of test data, which means the trained MLP can replace the coarse model.

After the accurate coarse model is built with mesh deformation, the surrogate model can be developed to represent the fine model response with different geometric and frequency parameters. Two mapping modules are added to the coarse model to develop the surrogate model. Unit mapping networks for the geometric parameters and frequency are established, keeping the model error consistent with the coarse model error. Then, the surrogate model is trained. Fast simulation of the fine model was operated in the HFSS simulator to generate training and test data with different geometric parameters. We use five levels (25 sets of data) of DOE to define the training data samples, and eight levels (64 sets of data) of DOE for the test data. Table 1 shows the specific values of the training and test data for the surrogate models. The test data were selected randomly from within the training range, and were not utilized for the training process. The surrogate model, including the coarse model and two mapping modules, was constructed and trained in Neuromodelerplus software. After the training process, the overall model, with a training error of 0.43% and a test error of 0.35%, was obtained. The total CPU time was about 1.66 h.

Comparisons of different parametric modeling approaches based on the network structure, training error, test error, training time, and CPU time are shown in Table 2; the training time includes the time to train the MLP for the coarse model and the surrogate model. ANN models [24] are directly trained without mapping networks. The ANN model trained with 25 sets of data (denoted as ANN model 1 using 50 hidden neurons) can learn the fine characteristics well, but the predictive ability of the model is poor. If we used 81 sets of data to develop an accurate ANN model (denoted as ANN model 2 using 60 hidden neurons), 4.43 h are needed to generate the training data, which greatly increases the modeling time. To prove the effectiveness of the mesh deformation technique, we develop an SM model using the existing SM method, which does not involve mesh deformation [4]. The coarse-mesh model with fewer mesh cells (about 4400 cells) is built in the HFSS software. Two MLPs, instead of the coarse model, are trained with 81 sets of data with 100 double-layered hidden neurons, and 169 sets of data with 100 double-layered hidden neurons, which are introduced into the existing SM model 1 and the existing SM model 2, respectively. As the response of the coarse model is non-continuous, instead, the MLP of the coarse model requires more data to express a higher nonlinearity. In addition, the existing SM model 2 containing high-nonlinear prior knowledge is easily overfitted during the training process, which makes modeling more difficult.

As can be seen from Table 2, when using less training data, the proposed model has a higher accuracy than the other two modeling methods within the training range. The coarse mesh model included in the surrogate model provides prior knowledge, which makes the proposed model more accurate than the ANN model. In addition, the increase in modeling time is minimal because the coarse model is built with inexpensive data. The mesh deformation technique guarantees that the response of the coarse model is continuous, which saves modeling time for the coarse model and improves the model accuracy. The training error and testing error of the proposed model are both less than 1%. When the errors are similar, the proposed method can save about 63% computational cost compared to the ANN modeling method. The proposed model achieves accuracy requirements, while utilizing fewer training data and less computer consumption within the training range.

**Figure 6:**
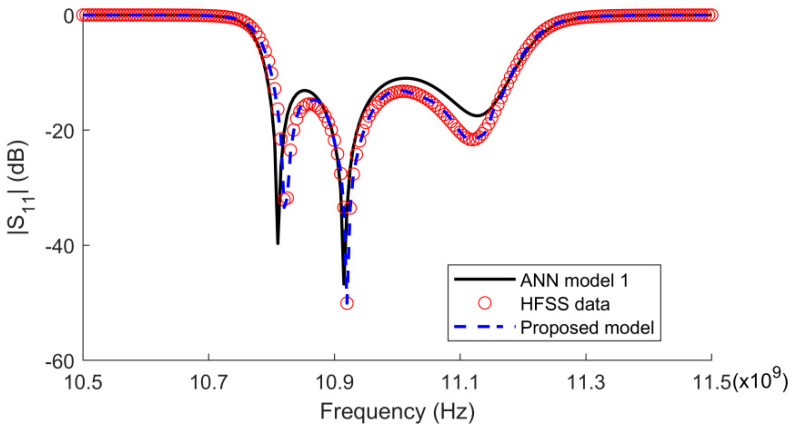
Comparison of the magnitude of $S_{11}$ for the three models.

For the four-pole waveguide filter with tuning element modeling, a comparison of the $S_{11}$ parameters for the fine model obtained in HFSS software, the ANN model trained with 25 sets of data, and the proposed surrogate model trained with 25 sets of data is shown in Figure 6. The results show that the proposed surrogate model can provide similar results to those of the commercial simulation software within the training range.

After training, the proposed surrogate model can accurately and quickly predict the response of the microwave components. The computational times for the proposed surrogate model and the model in HFSS software for different data are shown in Table 3. As the simulation data increase, the computation time of the software is very long, while the runtime of the proposed surrogate model increases very slightly. The greatest computing time costs in the surrogate model are in the training process and training data extraction. The operation efficiency of the proposed model obtaining new responses is very high, which greatly reduces the design cycle for new devices.

## 4. Conclusions

This paper proposes a mesh SM modeling method for microwave components that considers mesh deformation. When traditional equivalent circuits or empirical formulas are invalid, this method based on the coarse-mesh model can develop the surrogate model efficiently. The mesh deformation technique is embedded in the coarse model, which avoids discontinuous change in the electromagnetic response, and accelerates the modeling process. After the training process, the proposed surrogate model can accurately and quickly predict the EM response of the microwave component for different geometric and frequency parameters. The example of the four-pole waveguide filter verifies that the error of the proposed model is less than 1%, which is lower than that of the existing SM model. When obtaining the similar precision, the proposed model took 1.66 h, while the ANN model took 4.53 h, which proved that the modeling efficiency is improved greatly. The mesh SM modeling method with mesh deformation can develop the accurate model with less training data. In the future, some extrapolation methods can be introduced into the proposed mesh SM model to guarantee smoothness at the edge of the training range, improving the model accuracy inside and outside the training region.

## Figures and Tables

**Table 1:** The modeling data for the coarse and fine model of the four-pole waveguide filter.

Input Variables		Training Data Range		Test Data Range	
		Min	Max	Min	Max
Coarse model	h_1_ (mm)	3.2528	3.5952	3.3384	3.5096
	h_2_ (mm)	3.9102	4.3218	4.0131	4.2189
	h_3_ (mm)	3.4276	3.7884	3.5178	3.6982
Fine model	h_1_ (mm)	3.3213	3.5267	3.3042	3.5438
	h_2_ (mm)	3.9925	4.2394	3.9719	4.2601
	h_3_ (mm)	3.4998	3.7162	3.4817	3.7343

**Table 2:** Comparisons of the results of different modeling methods for the four-pole waveguide filter.

Modeling Method	Coarse Data	Fine Data	Training Error	Test Error	Fine Data Time	Coarse Data Time	Model Training Time	Total CPU Time
ANN model 1	0	25	0.47%	7.21%	1.43 h	0	101s	1.46 h
ANN model 2	0	81	0.48%	0.98%	4.43 h	0	353.8 s	4.53 h
Existing SM model 1	81	25	1.78%	2.45%	1.43 h	630 s	504.3 s	1.57 h
Existing SM model 2	169	25	1.37%	1.53%	4.43 h	1320 s	673.6 s	4.99 h
Proposed model	81	25	0.43%	0.35%	1.43 h	516 s	305.6 s	1.66 h

**Table 3:** CPU time comparison between the proposed model and HFSS software.

Number of Geometrical Parameters	CPU Time	
	Proposed Model	Simulation Using HFSS Software
1	1.66 h + 0.098 s	202.2 s
50	1.66 h + 0.3 s	2.52 h
100	1.66 h + 1.374 s	5.53 h

## Data Availability

The data are available from the corresponding author on reasonable request.
